# Formation of Protein Nanoparticles in Microdroplet Flow Reactors

**DOI:** 10.1021/acsnano.3c00107

**Published:** 2023-06-12

**Authors:** Qi Zhang, Zenon Toprakcioglu, Akhila K. Jayaram, Guangsheng Guo, Xiayan Wang, Tuomas P. J. Knowles

**Affiliations:** † Department of Chemistry, University of Cambridge, Lensfield Road, Cambridge CB2 1EW, U.K.; ‡ Cavendish Laboratory, Department of Physics, University of Cambridge, J J Thomson Avenue, Cambridge CB3 OHE, U.K.; § Center of Excellence for Environmental Safety and Biological Effects, Beijing Key Laboratory for Green Catalysis and Separation, Department of Chemistry, Beijing University of Technology, Beijing 100124, People’s Republic of China

**Keywords:** protein nanoparticles, droplet microfluidics, intracellular delivery, high-throughput nanoparticle formation, regenerated silk fibroin, Bovine serum albumin, beta-lactoglobulin

## Abstract

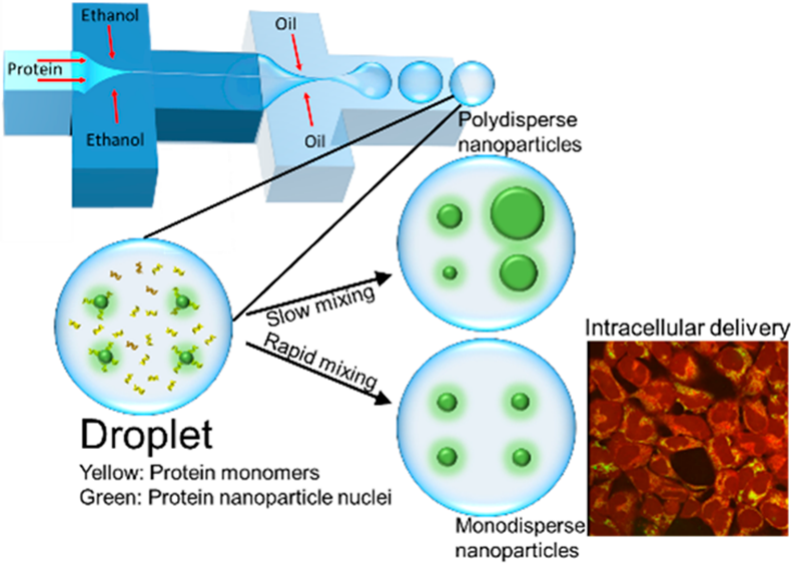

Nanoparticles are increasingly being used for biological applications, such as drug delivery and gene transfection. Different biological and bioinspired building blocks have been used for generating such particles, including lipids and synthetic polymers. Proteins are an attractive class of material for such applications due to their excellent biocompatibility, low immunogenicity, and self-assembly characteristics. Stable, controllable, and homogeneous formation of protein nanoparticles, which is key to successfully delivering cargo intracellularly, has been challenging to achieve using conventional methods. In order to address this issue, we employed droplet microfluidics and utilized the characteristic of rapid and continuous mixing within microdroplets in order to produce highly monodisperse protein nanoparticles. We exploit the naturally occurring vortex flows within microdroplets to prevent nanoparticle aggregation following nucleation, resulting in systematic control over the particle size and monodispersity. Through combination of simulation and experiment, we find that the internal vortex velocity within microdroplets determines the uniformity of the protein nanoparticles, and by varying parameters such as protein concentration and flow rates, we are able to finely tune nanoparticle dimensional properties. Finally, we show that our nanoparticles are highly biocompatible with HEK-293 cells, and through confocal microscopy, we determine that the nanoparticles fully enter into the cell with almost all cells containing them. Due to the high throughput of the method of production and the level of control afforded, we believe that the approach described in this study for generating monodisperse protein-based nanoparticles has the potential for intracellular drug delivery or for gene transfection in the future.

## Introduction

Nanoparticles have been extensively studied due to their unique size and biological, chemical, and physical properties, making them versatile in a wide range of applications.^1−4^ Such particles are ideal candidates for controlled delivery applications, as their size readily allows for cellular uptake.^5,6^ They have the potential to stabilize and carry cargo molecules, and as such, they are extremely attractive in the biomedical, pharmaceutical, cosmetic, food, and material-based industries. However, production of homogeneous and monodisperse nanoparticles with high encapsulation efficiencies, intracellular delivery rate, and rapid release of cargo molecules has proven difficult to achieve. There are currently a range of different methods for generating such nanoscale particles, including spray drying, ultrasonic emulsification solvent extraction, or even bulk emulsion and polymerization techniques.^7−10^ The issue with these techniques, however, is that precise control over size and monodispersity may remain problematic, and therefore regulating molecular release will be affected. A technique that can be used to overcome these difficulties and produce uniform, functional nanoparticles is microfluidics.^1,11−20^

Due to their high biocompatibility, biodegradability, versatility, low immunogenicity, and lack of cellular toxicity as natural biomolecules,^21,22^ protein nanoparticles are increasingly gaining interest in this context. Protein-based therapeutic approaches,^23^ which exploit individual soluble forms of proteins, have transformed drug discovery, and building on this success, there is increasing interest in exploring the assembly of protein building blocks into nanoscale carriers.^24−31^ There are currently multiple ways of synthesizing protein nanoparticles that include bulk desolvation,^7^ liquid–liquid phase separation^32^ and spray drying,^10^ which can produce protein nanoparticles with high throughput. However, these methods have poor control of the size and uniformity of protein nanoparticles. An alternative method of producing nanoparticles that achieves high control of particle size and monodispersity is nanofluidics.^33^ However, the combination of low flow rates being used and the high resistance within the nanoscale channels limits the possibility of high-throughput production of protein nanoparticles using the approach. Therefore, it is necessary to develop a systematic method for the high-throughput generation of uniform and stable protein-based nanoparticles.

In order to address these limitations, here, we use a droplet-microfluidic approach to generate protein nanoparticles within microdroplets. By utilizing the propensity of liquids to undergo rapid and continuous mixing inside droplets, we were able to produce highly monodisperse protein-based nanoparticles that could be used for intracellular delivery. Parameters such as aqueous and oil phase flow rates as well as protein concentration allowed us to control and regulate the size of protein nanoparticles. Moreover, by combining our results with finite element simulations, we elucidated the mechanism behind nanoparticle formation, and we describe how size and homogeneity of protein nanoparticles are affected as a function of parameter change. It was determined that the vortex velocity within the droplet is essential for preventing nanoparticles from aggregating following nucleation and is the reason behind uniform particle production. Finally, the smallest nanoparticles that we could form, 29 ± 11 nm, were used to investigate whether they would permeate the cellular membrane. Fluorescence and confocal microscopy were used to establish not only that are our nanoparticles highly biocompatible but that cellular uptake occurred for almost all cells. Moreover, stability tests were conducted, and it was determined that the protein nanoparticles remained stable even after 60 days. Furthermore, we show that this method can be generalized to form nanoparticles from a variety of proteins. In addition to reconstituted silk fibroin, nanoparticles of different sizes were generated by using this microfluidic approach from bovine serum albumin (BSA) and beta-lactoglobulin. Therefore, due to their stability, high level of monodispersity, and high throughput of production, this method of nanoparticle production is particularly promising for potential applications involving intracellular delivery of drugs or genes.

## Results and Discussion

**Figure 1:**
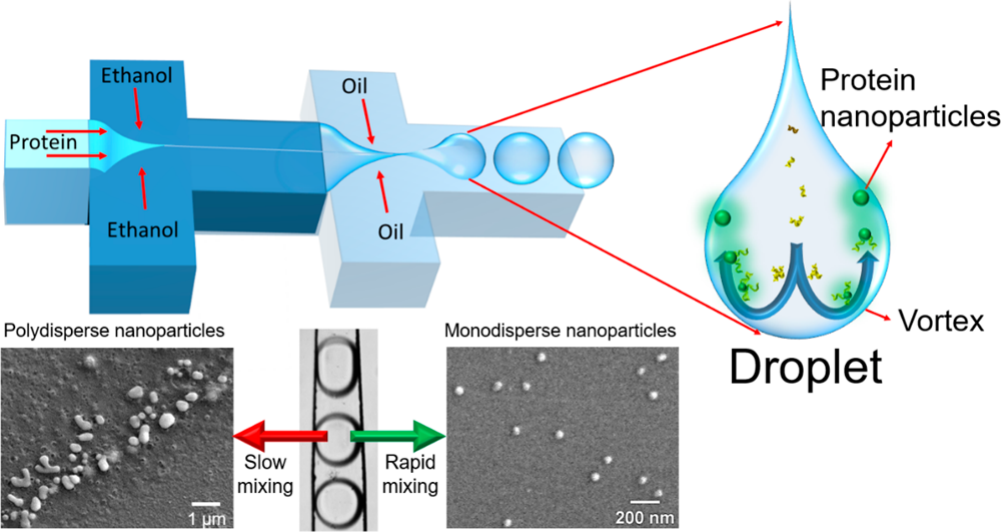
Schematic of the droplet-based microfluidic device used for producing protein nanoparticles. Once protein-containing droplets are formed, rapid mixing induced by the vortices within the droplets results in the formation of monodisperse protein nanoparticles.

In order to form monodisperse nanoparticles, microfluidics was employed. A droplet-microfluidic device, in which oil flows as the external phase, ethanol as the middle phase, and protein as the internal phase, was used (see schematic in Figure 1). First, ethanol reduces the solubility of protein molecules, effectively acting as a desolvating agent, resulting in protein nucleation and, ultimately, nanoparticle formation. The oil phase separates the reaction solution in the form of an aqueous droplet, and molecular interactions within the droplets become more rapid due to the high level of mixing within the droplets, which is instrumental in the formation of monodisperse nanoparticles. Compared with conventional 2D microfluidic chips, where fluids flow adjacent to each other and laminar flow does not allow for rapid mixing, in our device, rapid mixing within microdroplet reaction vessels allows for the formation of monodisperse and uniform nanoparticles (Figure 1). In this work, we explore the effects of three variables for the generation of protein nanoparticles: (1) we investigate the effect of protein concentration, (2) the effect of the oil phase flow rate on nanoparticle production, and (3), finally, how the ratio of the two aqueous phases with respect to each other affects nanoparticle production.

### Controlling Protein-Based Nanoparticle Formation for Optimal Conditions Exploration

**Figure 2:**
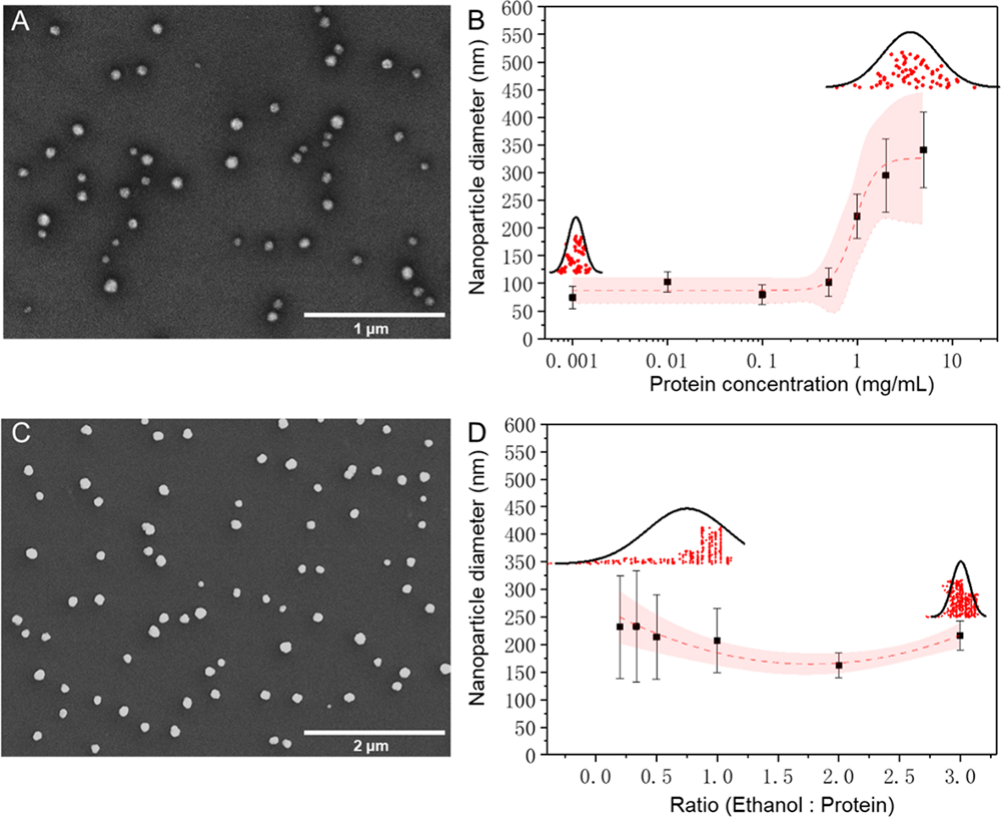
(A, B) Effect of protein concentration on nanoparticles. (A) SEM micrograph of a 0.5 mg/mL protein solution. (B) Graph of nanoparticle size as a function of protein concentration. (C, D) Effect of changing the ethanol-to-protein flow rate ratio on nanoparticle formation. (C) SEM micrograph of nanoparticles formed using a flow rate ratio of 2. (D) Graph of nanoparticle size as a function of the ethanol-to-protein flow rate ratio. The shaded regions represent the 95% confidence intervals for logarithmic models fitted to the individual points. Each point on the graphs indicates the average particle size for over 10,000 droplets. The distributions represent the concentration of statistical particle sizes under the most polydisperse and monodisperse experimental conditions (particles counted: *n* > 50).

We first varied the concentration of protein in order to investigate what effect this would have on the particle size. We found that when the concentration was less than 0.5 mg/mL, particle size did not change significantly and was around 80 nm, as confirmed with scanning electron microscopy (SEM), shown in Figure 2A. However, there is a critical concentration (0.5 mg/mL), beyond which the size of protein particles increased from 100 nm up to 350 nm. The effect of protein concentration on nanoparticle size is summarized in the graph in Figure 2B. Following this, we then investigated the effect that the ratio between the two aqueous phases has on the nanoparticles. It was determined that when we increased the ethanol-to-protein flow rate ratio, particle size decreased from 250 nm down to 160 nm, with better particle uniformity also being achieved. Moreover, we found that when this flow rate ratio was greater than 2, the size of protein particles started increasing (from 160 nm to 220 nm) and that particle distribution was not greatly affected. A typical SEM micrograph of protein nanoparticles with a uniform shape and spherical morphology at a flow rate ratio of 2 is shown in Figure 2C. The SEM results for all sample ratios are summarized in Figure S1, where it is evident that particle uniformity clearly depends on what flow rate ratio is used. The graph in Figure 2D shows how the nanoparticle size varies as a function of the ethanol-to-protein flow rate.

**Figure 3:**
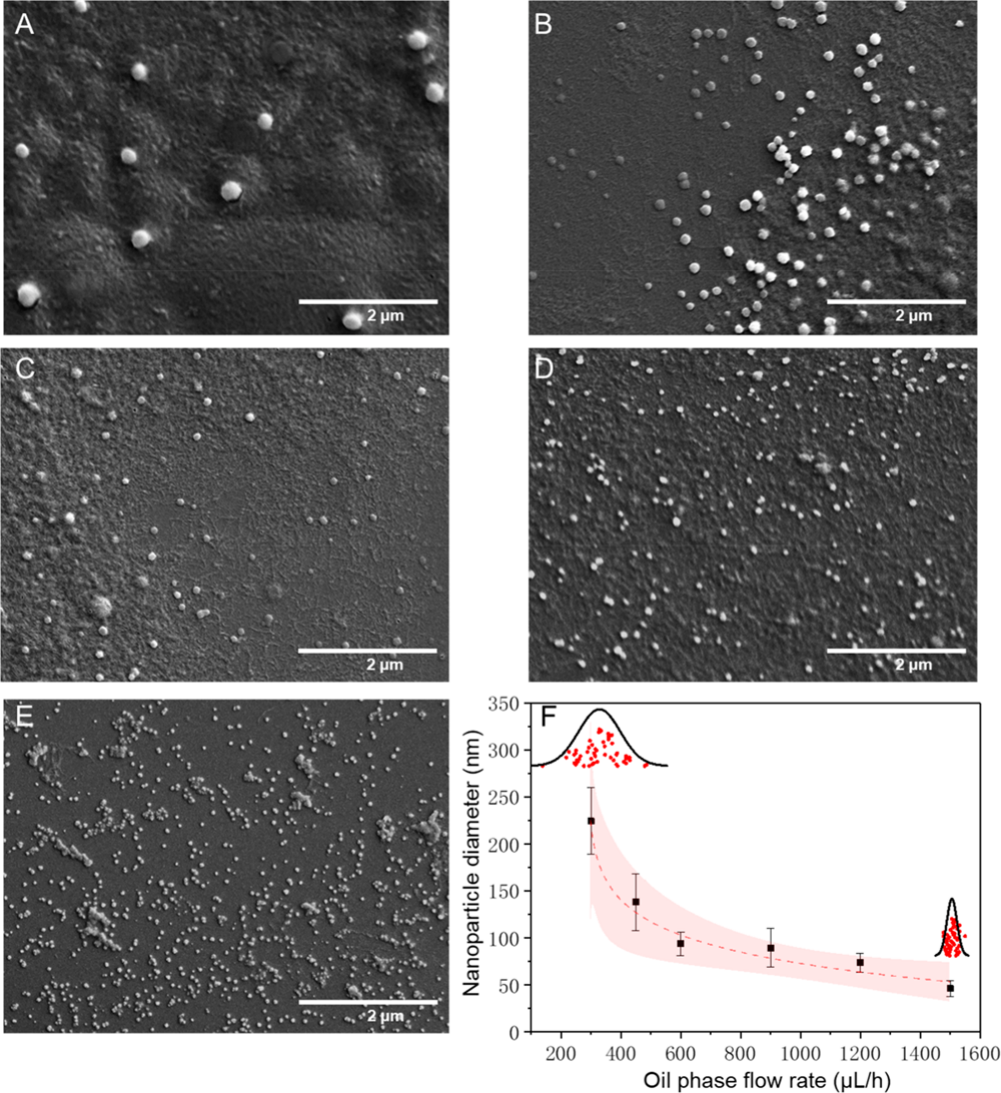
Effect of oil phase flow rate on protein nanoparticle formation. (A–E) SEM micrographs of protein nanoparticles formed when the oil phase flow rate was 300, 450, 600, 900, and 1500 μL/h, respectively. Aqueous phase flow rates were kept constant at 100 μL/h. (F) Graph of nanoparticle size as a function of oil phase flow rate. The shaded regions represent the 95% confidence intervals for logarithmic models fitted to the individual points. Each point on the graph indicates the average particle size for over 10,000 droplets. The distributions represent the concentration of statistical particle sizes under the most polydisperse and monodisperse experimental conditions (particles counted: *n* > 50).

Furthermore, we investigated how the flow rate of the external oil phase can affect the nanoparticle size. When increasing the flow rate of the oil phase, the diameter of the aqueous droplet decreased in an exponential manner, as shown in Figure S2. This was determined both experimentally and computationally. Interestingly, however, it was found that the oil phase flow rate also affects nanoparticle size distributions, the mechanism of which is discussed in the section below. The SEM results in Figure 3A–E show the changes of protein particle size and uniformity when the oil phase flow rate was 300, 450, 600, 900, and 1500 μL/h, respectively, while the flow rate of the two aqueous phases was kept constant at 100 μL/h. We found that the size of protein nanoparticles gradually decreases from 225 nm to 50 nm, while particle uniformity also gradually increased, as can be seen from the graph in Figure 3F.

### Mechanistic Explanation behind Size and Monodispersity of Protein Nanoparticle Formation

Through the combination of finite element simulation software (COMSOL) and high-speed camera imaging, we explain the mechanism behind nanoparticle formation within microdroplets. The addition of ethanol to a protein solution affects the solubility of protein molecules, making it easier to desolvate protein molecules and thus initiating and facilitating nucleation, which ultimately leads to nanoparticle formation. From the graph in Figure 2B, which shows the relationship between nanoparticle size as a function of protein concentration, it is clear that a critical concentration exists (namely, 0.5 mg/mL) beyond which nanoparticle size increases as protein concentration is increased. When a higher protein concentration is used, each microdroplet consists of more protein molecules, and thus, when nuclei are formed within the microdroplets, there are more free protein monomers to help nuclear growth, which results in the production of larger nanoparticles. COMSOL simulations were conducted, which confirm this, the results of which are shown in Figure S3.

**Figure 4:**
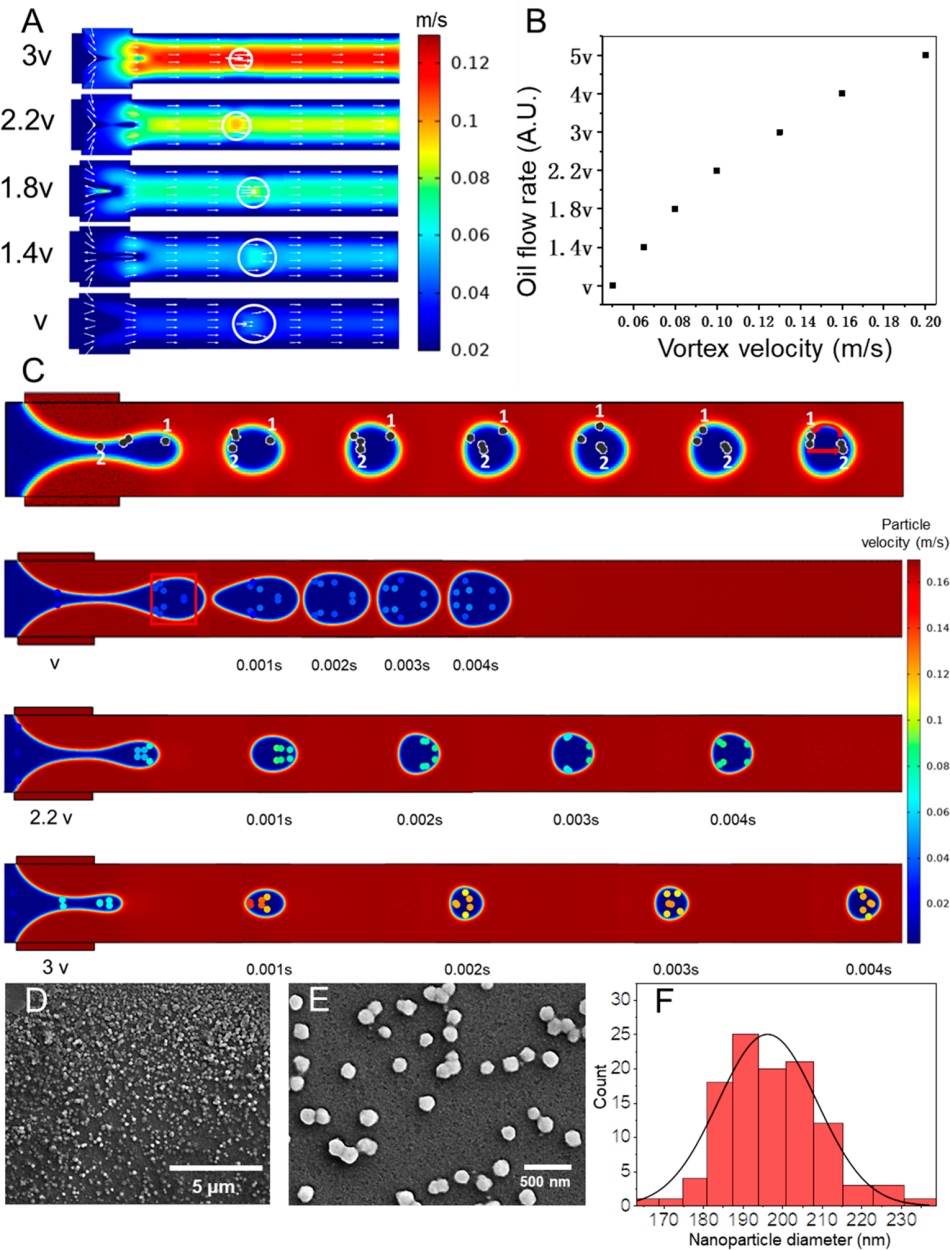
Finite element simulation results showing the velocity field and molecular tracking in the moving droplets. (A) Simulation result showing the different velocity distributions in the droplet as a function of changing the oil phase flow rate. The white circle corresponds to the edge of the droplet, and the arrows correspond to the flow direction. (B) Graph of droplet velocity field as a function of oil phase flow rate. (C) Simulation results showing nanoparticles’ movement within droplets. Nanoparticles, which are numbered, can clearly be seen moving as the droplet tumbles. Time-frame tracking of molecular movement within the droplets for different oil phase flow rates is shown. (D, E) SEM micrographs of nanoparticles with a 10 mg/mL protein concentration, a 9:1 ethanol-to-protein ratio, and an oil phase flow rate of 1500 μL/h and (F) the corresponding particle size distribution (particles counted: *n* > 100).

It was found that the size of protein nanoparticles decreased when increasing the oil phase flow rate but also that nanoparticles became more monodisperse. In order to explain this observation, simulations and experiments were conducted. Droplet formation was simulated under different oil phase flow rates, and small spheres (which represent molecules/nanoparticles) were added to the simulation so that we could monitor their trajectories as droplets were formed. Figure 4A shows the velocity field distribution of droplet generation at different oil phase flow rates. It can be seen from the figure that the velocity field in the droplet increases as the oil phase flow rate is increased; the graphical result of this is summarized in Figure 4B. The simulation results show that the higher the oil phase flow rate, the more intense the movement of the molecules within the droplet per unit time, with the majority of the molecules moving in a circular manner along the vortex field within the droplet (Figure 4C, top panel). This rapid movement, which is shown in the middle and bottom panels in Figure 4C, is the reason we obtain better nanoparticle monodispersity at larger oil phase flow rates. By rapidly and continuously mixing the ethanol with the protein phase, the ethanol effectively modifies the Flory parameter of the system to take it into the bad solvent regime, thus forcing the protein molecules to come together and initiating nucleation of nanoparticles. In order words, the nucleation is triggered by the fact that the addition of ethanol decreases the solubility of the protein and thus generates a supersaturated solution. Under such conditions, attractive intermolecular forces, predominantly hydrogen bonds and electrostatic and hydrophobic forces, drive self-assembly and bring molecules together to form clusters which can then grow into nanoparticles. The propensity of protein molecules to form supramolecular structures through self-assembly, stabilized by such interactions, has been exploited in order to form a rich diversity of protein-based materials in other contexts.^7−9,15,16,31^ In our microdroplet reactor, because of the enhanced mixing, protein nuclei and free monomers are homogeneously distributed within the microdroplet, which allows for increased uniform growth of the nanoparticles. Effectively, when rapidly mixing, each nucleation site has an equal probability of coming into contact with the same number of protein monomers, which is not necessarily the case when the system is poorly mixed and nanoparticle growth is predominantly diffusion limited. This phenomenon can be compared to producing nanoparticles in bulk, where there is poor and limited mixing and molecular diffusion plays an integral part. As can be seen in Figure S4, when performing this mixing experiment in bulk, huge particles are formed with massive polydispersity. This shows how important rapid and continuous mixing is in ensuring control over nanoparticle size, monodispersity, and uniformity.

Furthermore, in order to gain insights into why we obtained a higher degree of nanoparticle polydispersity when using a higher protein concentration, COMSOL simulations were conducted. Differences in the protein concentration were simulated by altering the viscosity of the solution. From the results we can see that when we simulate the velocity field in a droplet with the same oil phase flow rate but different viscosity in the protein phase, we find that the higher the viscosity, the larger the droplet size and the smaller the vortex velocity within the droplet. That is to say that when the protein concentration is increased and therefore the viscosity of the aqueous phase is higher, the vortex velocity within the droplet is lower, and so there is poorer mixing. These results are shown in Figure S5. This prompts us to conclude that particle uniformity is worse for higher protein concentration solutions due to poorer and slower mixing.

In order to understand the decrease in polydispersity when the ethanol-to-protein flow rate is increased (Figure 2D), high-speed camera images were taken. The protein phase was tagged with a dye, methylene blue, so that it could be visualized with a bright field microscope. As shown in Figure S6, when the ethanol-to-protein ratio is increased, the laminar flow of the ethanol phase with the protein phase limits mixing to the central region of the droplets. As discussed earlier, the velocity vortex within the droplet is larger, closer to the center of the droplet. That is to say, the closer molecules are to the center of the droplet, the faster they mix. Therefore, when the ethanol-to-protein flow rate ratio is increased and protein molecules are mostly confined to the center of the droplet, they undergo better mixing in this local environment, which explains the increase in monodispersity.

Moreover, in order to verify that rapid and continuous mixing is essential for control over particle size and polydispersity, we prepared nanoparticles using a high protein concentration (10 mg/mL). As shown and discussed previously, high protein concentration experiments yielded nanoparticles that had large sizes (350 nm) and quite high polydispersity. However, even if a 10 mg/mL solution is used, by flowing the oil phase at a flow rate of 1500 L/h and therefore creating a rapid mixing environment, the protein nanoparticles produced were not only smaller but extremely monodisperse, 196 ± 13 nm (Figure 4D–F), which further corroborates our mechanistic explanation behind nanoparticle generation.

**Figure 5:**
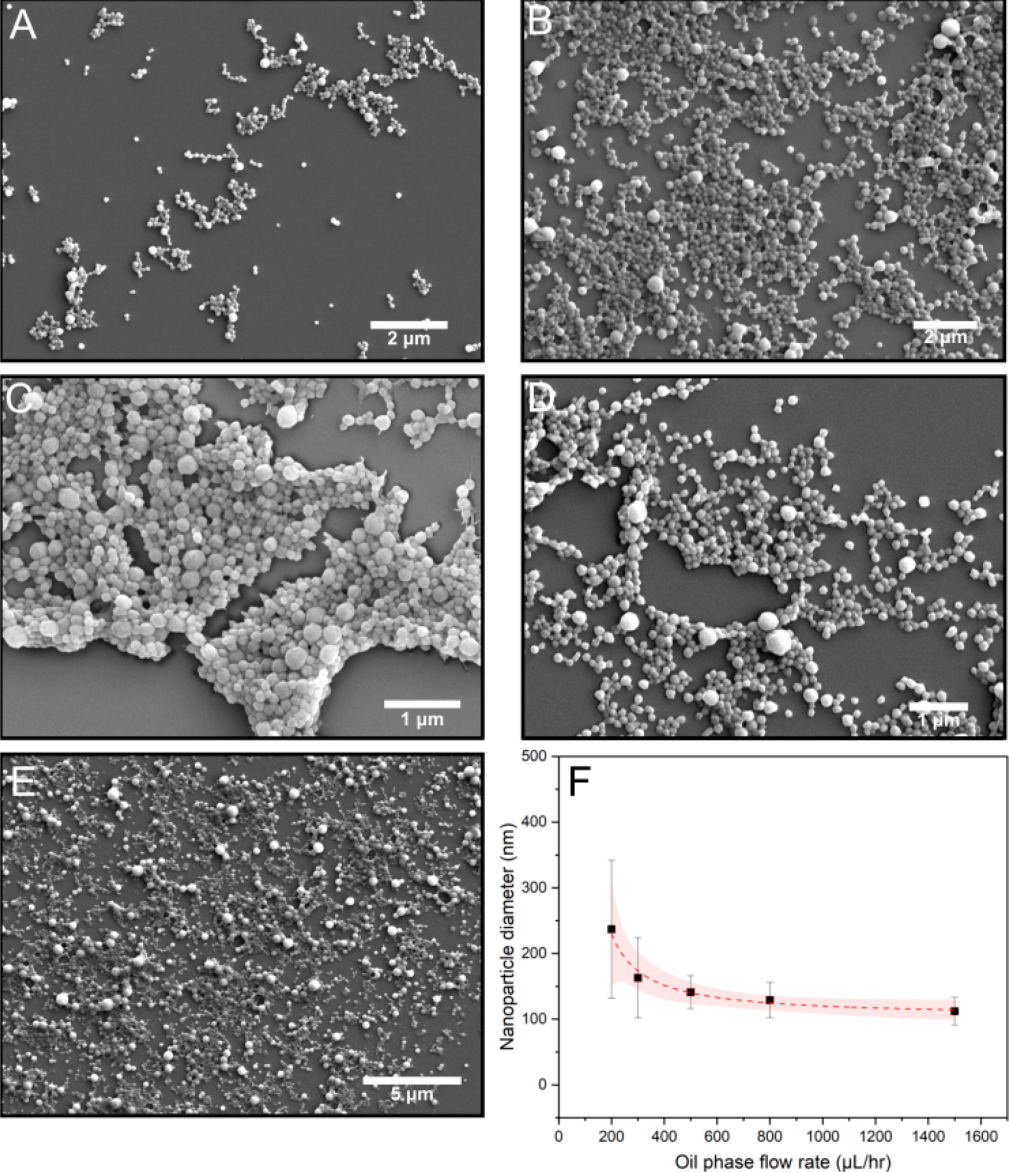
Effect of oil phase flow rate on BSA protein nanoparticle formation. (A–E) SEM micrographs of protein nanoparticles formed when the oil phase flow rate is varied. (F) Graph of nanoparticle size as a function of the oil phase flow rate. The shaded regions represent the 95% confidence intervals for logarithmic models fitted to the individual points. Each point on the graph indicates the average particle size for over 10,000 droplets (particles counted: *n* > 50).

In order to show that our microfluidic approach can be applied for the general production of nanoparticles, two additional proteins were investigated. Nanoparticles were generated using the same microfluidic method for both BSA and beta-lactoglobulin. As conducted earlier, we investigated how the flow rate of the external oil phase can affect the nanoparticle size. Again we found that when increasing the flow rate of the oil phase, we generated more monodisperse and smaller nanoparticles. For BSA, we were able to form nanoparticles ranging from 250 down to 100 nm by varying the oil phase flow rate from 200 to 1500 μL/h, respectively. This is shown in Figure 5F, while typical SEM micrographs of nanoparticles formed using different oil phase flow rates are shown in Figure 5A–E. Finally, we were also able to form nanoparticles from beta-lactoglobulin, with sizes ranging from 250 to 50 nm for the same oil phase flow rate range. These results are summarized in Figure S7.

### Intracellular Uptake of Nanoparticles

**Figure 6:**
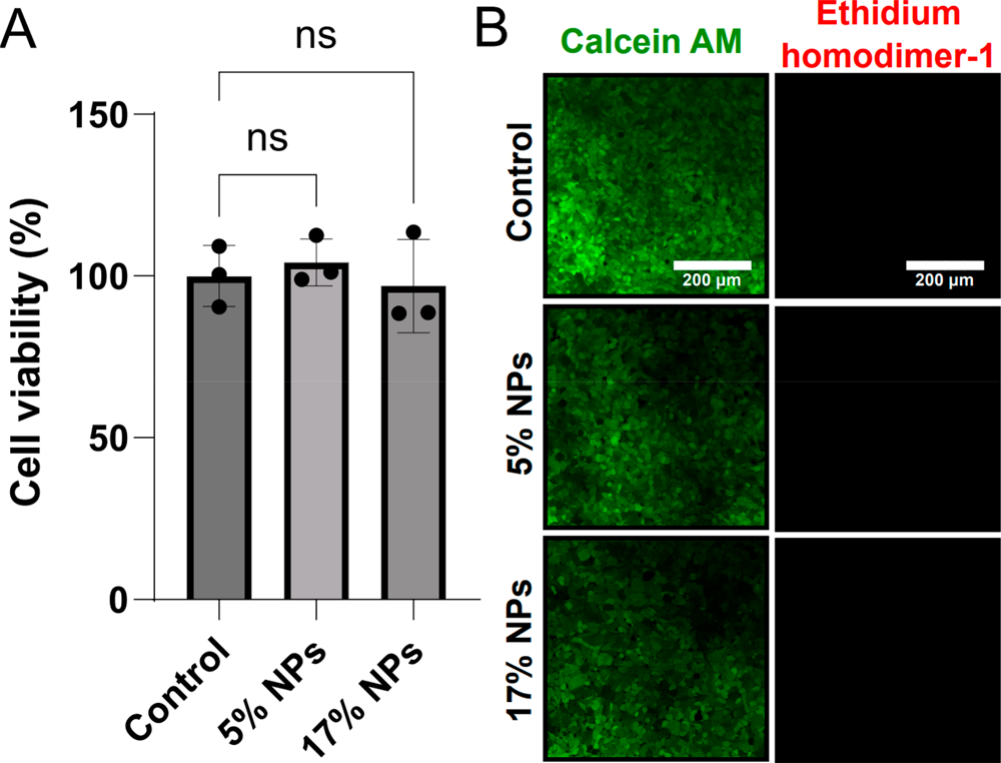
Biocompatibility of the protein-based nanoparticles with HEK-293 cells. (A) MTT cell viability analysis. The cytotoxicity and viability of HEK-293 cells with and without nanoparticles were assessed by an MTT assay following the overnight incubation of the cells with the silk nanoparticles. The data show the mean ± SEM of at least *n* = 3 individual experiments. A one-way ANOVA test was conducted, and in all cases, no significant difference in viability between the control and the different nanoparticle samples was observed. n.s. = not significant. (B) HEK-293 cell viability analysis with nanoparticles and controls following an overnight incubation with and without nanoparticles. This was carried out using a fluorescence-based live–dead staining assay containing calcein AM (which stained live cells) and propidium iodide (which stained dead cells). The scale bar for all microscopy images was 200 μm.

In order to establish whether silk nanoparticles could be used for intracellular delivery, we first looked into their compatibility with mammalian cell lines. Human embryonic kidney (HEK-293) cells were used to this effect, and an MTT-based viability assay was used to evaluate the degree of biocompatibility. The cells were grown in 96-well plates and left with and without the presence of nanoparticles overnight. Optimal conditions for forming protein nanoparticles (an oil phase flow rate of 1500 μL/h, a protein concentration of 0.1 mg/mL, and an ethanol-to-protein flow rate ratio of 2) were used. The nanoparticles were characterized via electron microscopy (SEM and TEM) and were found to be 29 ± 11 nm, as shown in Figure S8. Two different nanoparticle concentrations (5% solution and 17% solution) were tested with the HEK cells. In both cases, cell viability was not affected by the presence of the silk-based nanoparticles, as can be seen in Figure 6A, with both samples showing complete biocompatibility. At least 3 individual experiments were conducted, while a one-way ANOVA test showed that in all cases no significant difference in the viability between the control and the different nanoparticle samples was seen. n.s. means not significant. Additionally, a live/dead analysis of the HEK-293 cells, which were again cocultured with the two nanoparticle concentrations, yielded the same results. Calcein AM staining (which stains and indicates live cells) and ethidium homodimer-1 (which stains and indicates dead cells) staining were conducted (Figure 6B). Top panels show typical images of HEK-293 controls, while the middle and bottom panels correspond to cells incubated with 5% and 17% nanoparticles, respectively. From these images, it is clear that there is minimal cell death in the presence of our nanoparticles. Taken together, these data show the excellent biocompatibility of our protein nanoparticles and suggest that these nanoparticles could be used for intracellular delivery.

Moreover, in order to show that differently sized nanoparticles do not affect cellular biocompatibility, we performed an MTT viability assay on both 250 and 350 nm sized silk nanoparticles. In both cases, we found an extremely high cellular biocompatibility. Additionally, we performed the same biocompatibility assay for concentrated samples (17%), of different sized BSA and beta-lactoglobulin nanoparticles, where it was also determined that the cellular viability was extremely high. All these results are summarized in Figure S9. The data show the mean ± SEM of at least 3 individual experiments. A one-way ANOVA test was conducted, and it was found that in all cases, no significant difference in the viability between the control and the different nanoparticle samples was seen. n.s. means not significant.

**Figure 7:**
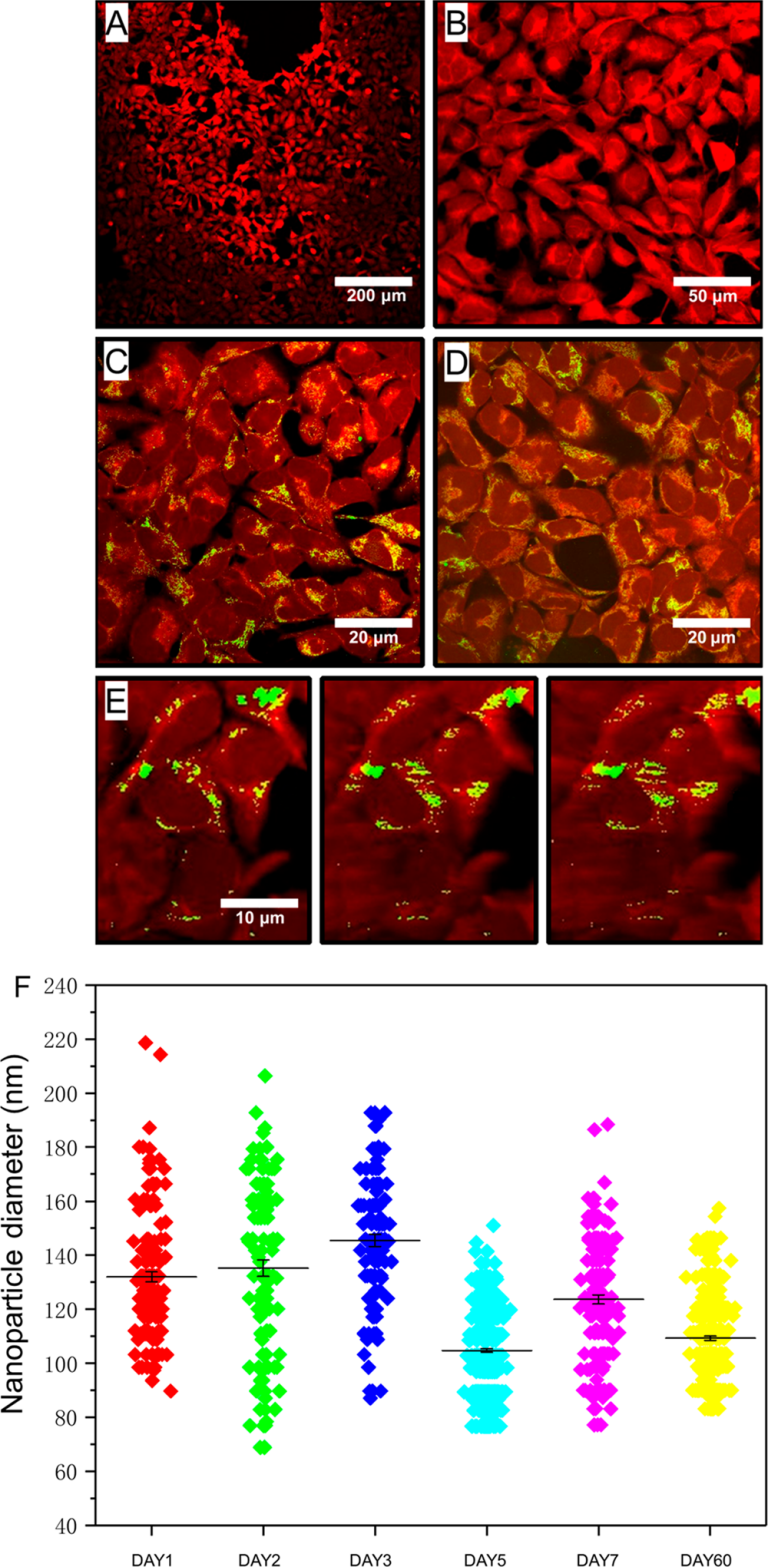
Analysis of protein-based nanoparticle uptake by HEK-293 cells. (A–E) Confocal microscopy images of HEK-293 cells with and without nanoparticles. Cells are shown in red, while nanoparticles are shown in green. (A, B) Fluorescence microscopy images of the HEK-293 cells without any nanoparticles. (C, D) Confocal microscopy images of HEK-293 cells in the presence of two nanoparticle concentrations, 5% and 17%, respectively. (E) 3D reconstruction of a few HEK-293 cells imaged at different angles to show that the cells have taken up the nanoparticles. (F) The size distribution of nanoparticles after being stored at room temperature for up to 60 days (particles counted: *n* > 100). The scale bars are 200, 50, 20, and 10 μm for panels (A), (B), (C, D), and (E), respectively.

To determine whether the nanoparticles produced using this droplet-microfluidic approach could potentially be used for intracellular applications, we conducted intracellular uptake studies. In order to establish whether nanoparticles were able to penetrate the cellular membrane, HEK-293 cells were stained with CellTracker Violet BMQC dye (λ_ex_ = 415 nm and λ_em_ = 516 nm), while the nanoparticles were tagged via a conjugation process with Atto 488. Cells and nanoparticles were cocultured overnight. Again, two different nanoparticle concentrations were used to investigate whether cellular penetration could be achieved. Before imaging, the cells were washed to remove any excess nanoparticles, and confocal microscopy was used to determine the degree of penetration. Nanoparticles are shown in green, while cells are depicted in red (Figure 7).

As predicted, cell controls containing no nanoparticles showed no fluorescence signal at an emission wavelength of 520 nm, which is the characteristic peak for the nanoparticles tagged with the dye (Figure 7A,B). However, upon addition of the nanoparticles, green areas can be seen, which clearly overlap with cells (Figure 7C–E). Particle uptake by cells was observed for both nanoparticle concentrations (5% and 17%, Figure 6C and 6D, respectively). Furthermore, from the confocal images, it can be seen that not only did we have nanoparticle uptake but almost all cells contain nanoparticles. We can therefore conclude that the intracellular uptake of these nanoparticles is almost 100%. Lastly, in order to determine whether the nanoparticles just adhere to the cell membrane or whether they are within the cell, a three-dimensional (3D) reconstruction of a few single cells was performed. As seen from the images taken at different angles, it can be seen that both nanoparticle solutions do, in fact, penetrate into the cells.

Finally, the stability of the protein nanoparticles was measured over time by observing whether the protein nanoparticles underwent morphological changes in size and homogeneity. Particles were left at ambient conditions for up to a week, and SEM micrographs were taken after 1, 2, 3, 5, 7, and 60 days. The size distribution over time results is shown in Figure S10 with the corresponding SEM data summarized in Figure 7F. It is clear that the protein nanoparticles exhibited excellent stability even after 2 months formation, with nanoparticle sizes and monodispersity remaining constant over time. Furthermore, as can be seen in Figure S10, the particle morphology was smooth with a spherical geometry. These experiments reveal that our nanoparticles can potentially be used for long-term applications and do not require any special storage conditions. Moreover, the stability of the nanoparticles over the same time period was monitored using dynamic light scattering (DLS). The results corroborate what was determined using SEM and reveal that there are no observable changes in the nanoparticle sizes as a function of time (Figure S11).

In order to show that our nanoparticles can remain stable even in a human environment, we sought to mimic such a system using a microfluidic approach. Nanoparticles were passed through microfluidic channels, and the sizes of the particles were measured both before and after passing through the channels. No significant differences were measured between the samples, showing that the impact of a microfluidic environment is minimal on our protein nanoparticles, and they remain stable when traversing through a microfluidic channel. The DLS data of the nanoparticles before and after passing through the microfluidic channel are shown in Figure S12.

## Conclusions

In summary, we used a droplet-microfluidic device as a tool to form protein nanoparticles with control over size and uniformity. By utilizing the propensity of liquids to undergo rapid and continuous mixing within microdroplets, we were able to use these aqueous droplets as reaction vessels and generate nanoparticles within them in an extremely high-throughput manner. Ethanol, a known desolvating agent, was mixed with silk protein in order to initiate nanoparticle formation. It was found that microdroplet internal vortex velocity determines the degree of molecular interactions and consequently can prevent nanoparticle aggregation following nucleation, resulting in control over particle uniformity, with poor mixing leading to more polydisperse nanoparticles, whereas rapid mixing led to higher particle monodispersity. Compared with alternative methods of generating nanoparticles, such as using co-flow microfluidic devices^31^ or nanofluidic droplet makers,^33,34^ or even using conventional bulk approaches^35^ (Figure S4), the controllability, uniformity, and size range of the nanoparticles generated using our droplet-microfluidic strategy show significant improvements, including a massive increase over particle throughput, especially when compared to the majority of other microfluidic techniques. Moreover, given the high level of biocompatibility and low toxicity toward mammalian cells, and coupled with the ability of our nanoparticles to enter into the cell, we believe that this method of generating nanoparticles has the potential for a variety of biomedical applications. Additionally, we show the robustness of this droplet-microfluidic approach, as it can be utilized to generate nanoparticles from a variety of proteins, including silk fibroin, BSA, and beta-lactoglobulin. Furthermore, by integrating RNA or drug molecules in the aqueous phase, the protein nanoparticles prepared using this microfluidic method have the potential to be used in biotechnological fields such as intracellular drug delivery or for transgenic delivery.^36^

## Methods

### Fabrication of the Microfluidic Chip

The master was fabricated by spin coating a 25 μm thick negative photoresist (SU-8 3025, MicroChem) onto a silicon wafer and then soft baking at 95 °C for 15 min. The mask was placed onto the wafer, exposed under UV light, and postbaked at 95 °C for 5 min. Then, the master was developed in propylene glycol methyl ether acetate (PGMEA; Sigma-Aldrich) to remove any excess photoresist. Microfluidic devices were fabricated using a 10:1 ratio of prepolymer PDMS to curing agent (Sylgard 184, DowCorning, Midland, MI, USA) and cured for 3 h at 65 °C. The PDMS was cut by a knife and peeled off the masters, and holes of 0.75 mm were punched on the PDMS molded from the master. Following this, the PDMS was treated with a plasma bonder (Diener Electronic, Ebhausen, Germany), and it was bonded on a glass slide with the channels facing downward. The device was baked at 65 °C for 24 h to ensure successful bonding. Finally, the device was injected with Aquapel solution in order to make the channels hydrophobic.

### Droplet Formation

The flow rates within the channels were controlled using neMESYS syringe pumps (Cetoni, Korbussen, Germany). For water-in-oil droplets, the dispersed phase was a protein solution, while fluorinated oil (Fluorinert FC-40, Sigma-Aldrich) containing 2% w/w fluorosurfactant (RAN Biotechnologies) was used as the continuous phase. Protein stock solutions of 0.001, 0.01, 0.1, 1, 2, and 5 mg/mL were prepared for reconstituted silk fibroin (purification process mentioned below). Mikrotron cameras were used for high-speed brightfield imaging and protein stains by methylene blue hydrate (BCBN8454 V, Fluka Analytical) for the mechanism explanation experiment.

### Electron Microscopy

For SEM, the sample was mounted onto a silicon wafer, and a 10 nm platinum layer was then sputter-coated onto it. Images were obtained using a TESCAN MIRA3 FEG-SEM at 5 kV. For transmission electron microscopy (TEM), the sample was mounted onto a carbon grid and stained with uranyl acetate. Images were acquired using a Tecnai G2 80 to 200 kV TEM.

### Confocal Microscopy

A confocal microscope (Leica TCS SP5 X) was used for imaging cell samples. A diode 405 and an argon laser were used for violet and green excitation, respectively. The 3D images were reconstructed by using ImageJ software.

### Nanogel Formation and De-emulsification

Following formation, microdroplets were collected and left at room temperature for a couple of hours. Nanoparticles were then extracted from the droplets by de-emulsification. Microdroplets were first washed with an FC-40 solution three times. Following this, 20% 1*H*,1*H*,2*H*,2*H*-perfluoro-octanol (Alfa Aesar) was added to the emulsion as well as an equal amount of deionized water. The samples were then centrifuged at 1000 rpm for 2 min, which resulted in full separation of the phases. This de-emulsification process was repeated three times before the final solution containing the nanoparticles was collected.

### Silk Fibroin Preparation and Purification

Silk fibroin was obtained from *Bombyx mori* silk cocoons [Mindsets (UK) Limited] by a well-established protocol.^37^ The cocoons were cut into small pieces and boiled for 30 min in a beaker containing 0.02 M sodium carbonate solution. This ensured that the sericin present in the silk fibers dissolved, while the insoluble silk pigments were retained. The silk fibroin was then removed from the beaker, rinsed three times with cold water, and left to dry overnight. Then 9.3 M lithium bromide was prepared by preparing a 20% (w/v) solution (i.e., a 1:4 ratio of silk fibroin to lithium bromide) to dissolve the dried silk fibroin, and the mixture was left in an oven at 60 °C for 4 h. To remove the LiBr, the silk–LiBr solution was placed in a 3 kDa dialysis tube and then was placed in a beaker containing ultrapure water. To ensure mixing, a large magnetic stirring rod was used, and the beaker was placed on the magnetic stirring plate. The water was changed a total of six times over 48 h. Finally, the silk protein solution was removed from the dialysis tubing and centrifuged at 9000 rpm for 20 min at 4 °C to remove any impurities. Centrifuged twice, the final product was stored in an Eppendorf tube in a refrigerator at 4 °C. In order to prevent gelation, all experiments were carried out within 2 weeks of extraction and purification of silk fibroin.

## Supporting Information Available

Additional materials and methods, cell culture of HEK-293 cells, cytotoxicity and cell proliferation using the MTT assay on HEK-293 cells, and viability analysis of HEK-293 cells (PDF)
