# Supplementary material for: Formation of Protein Nanoparticles in Microdroplet Flow Reactors
Source: ACS Nano. 2023 Jun 12;17(12):11335–44. doi: 10.1021/acsnano.3c00107 (PMC10311583; doi:10.1021/acsnano.3c00107)
Supplement: Supplementary file 1 — nn3c00107_si_001.pdf [file nn3c00107_si_001.pdf]

Supporting Information

# Formation of Protein Nanoparticles in Microdroplet Flow Reactors

Qi Zhang[ac*], Zenon Toprakcioglu[a*], Akhila K. Jayaram[ab], Guangsheng Guo[c,3], Xiayan Wang[c,2] and Tuomas P. J. Knowles[ab,1]

[a] Department of Chemistry, University of Cambridge, Lensfield Road, Cambridge CB2 1EW, UK.

[b] Cavendish Laboratory, Department of Physics, University of Cambridge, J J Thomson Avenue, Cambridge CB3 OHE, UK.

[c] Center of Excellence for Environmental Safety and Biological Effects, Beijing Key Laboratory for Green Catalysis and Separation, Department of Chemistry, Beijing University of Technology, Beijing 100124, PR China

**Table of Contents**

1. Experimental methods
2. Supplementary figures
   Figure S1~Figure S12

## 1. Experimental methods

**Cell culture of HEK-293 cells**. Human embryonic kidney 293 (HEK-293) cells were cultured in flasks (50cm2) at 37oC and with 5% CO2. Advanced Dulbecco’s modified Eagle medium (DMEM; TFS) was used along with the addition of 10% fetal bovine serum (Merck). Additionally, 50 U/mL penicillin, 50 µg/mL streptomycin (TFS), 50 µg/mL gentamicin (TFS) and 1\% (v/v) GlutaMax (TFS) were also added to the culture.

**Cytotoxicity and cell proliferation using MTT Assay on HEK-293 cells**. In order to determine the biocompatibility of the protein nanoparticles with mammalian cells, an MTT assay was performed on the HEK-293 cells. In brief, cells were incubated with the nanoparticles in a 96-well plate for 24h at 37oC and 5% CO2 at a seed concentration of approximately 105 cells per well. Following the 24h incubation, 10 µL of MTT (3-[4,5-dimethylthiazol-2-yl]-2,5-diphenyltetrazolium bromide) labelling reagent Merck) was added to each well with an incubation period of 4 hours. 100 µL of the solubilisation solution was then added to each well and a further overnight incubation was performed at 37oC and 5% CO2. Finally, the absorbance measurement of the resulting solution was conducted at 595 nm using a FLUOstar Omega microplate reader (BMG Labtech).

**Viability analysis of HEK-293 cells**. HEK-293 cell viability was determined using a LIVE/DEAD™ Viability/Cytotoxicity Kit (Invitrogen). Approximately 105 cells were seeded per well, and two nanoparticle concentrations were added (a 5% and 17% solution). The cells were incubated with the nanoparticles for 24 hours. 5 µL of calcein AM (Component A) and 20 µL of ethidium homodimer-1 (Component B) were added to 10 mL Dulbecco's PBS to create a stock solution. 100-200 µL of the stock dye solution was added to each well and the cells were observed using confocal microscopy (Leica TCS SP8 inverted confocal microscope).

## 2. Supplementary figures

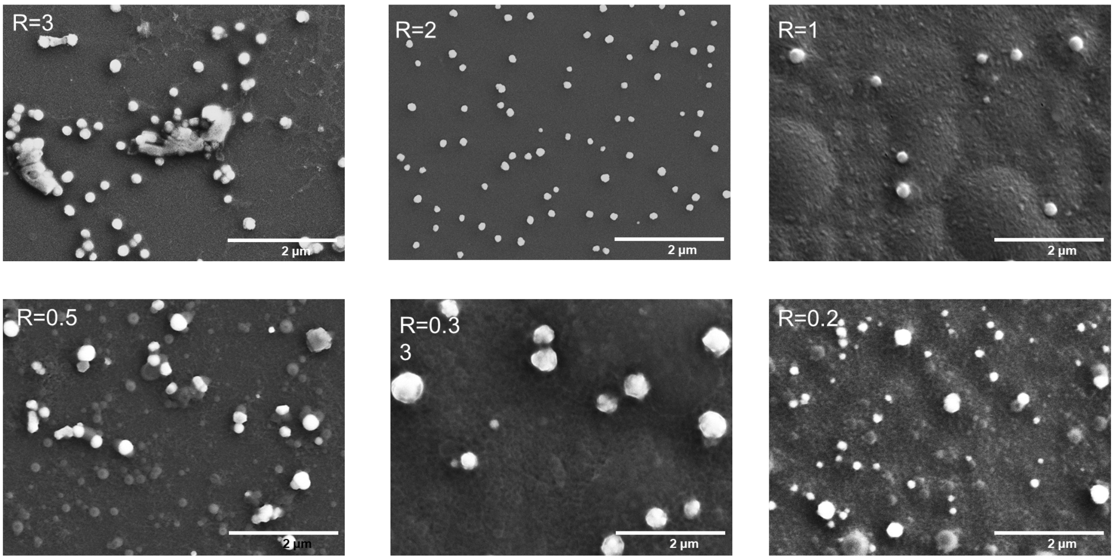


Figure S1. SEM micrographs of protein nanoparticles formed using a different Ethanol-to-Protein ratio which is denoted by R.

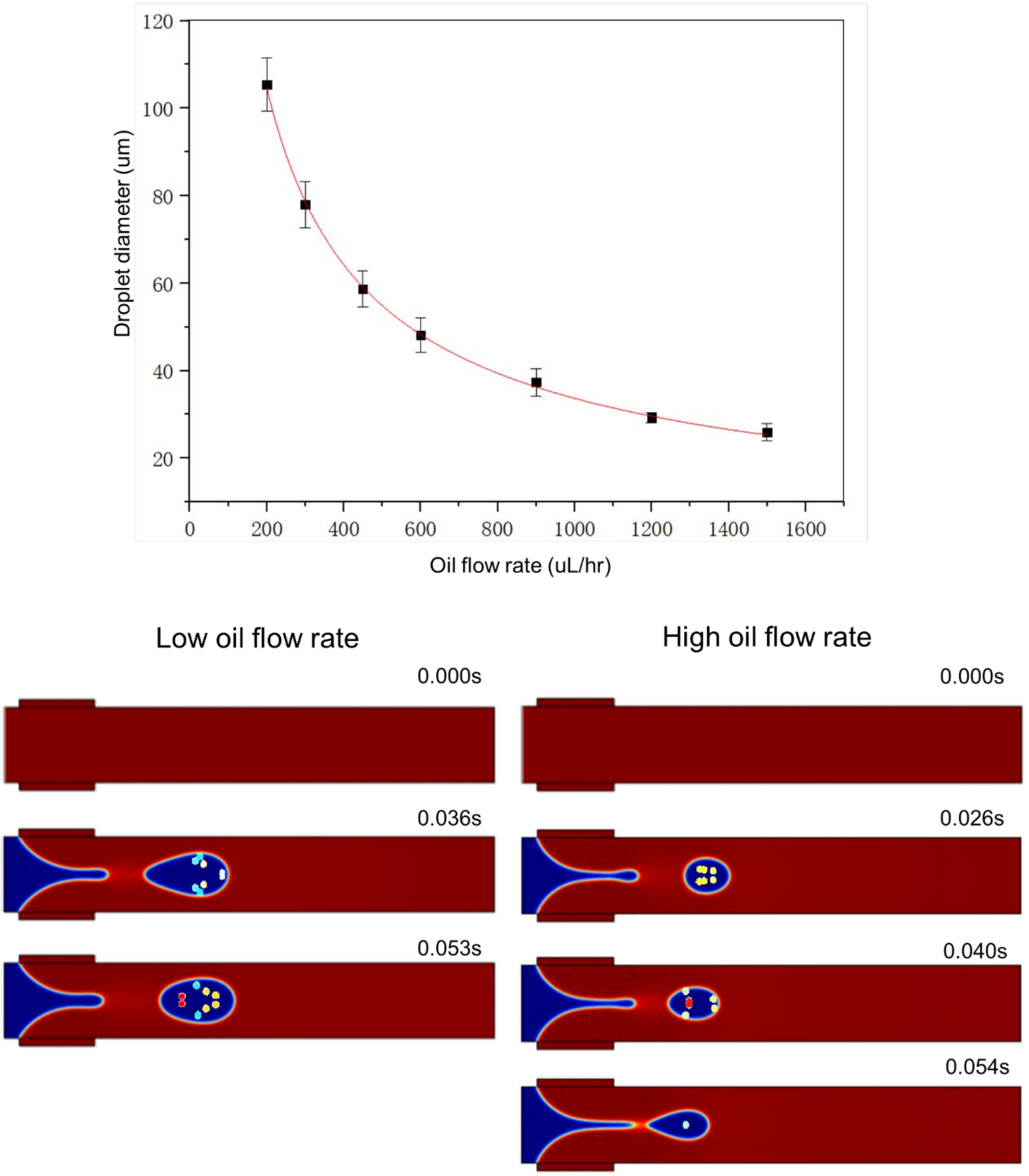


Figure S2. Graph showing droplet size of the oil phase flow rate both experimentally (up) and computationally (bottom). The lower the flow rate, the bigger the pico-droplets which contains more protein molecules.

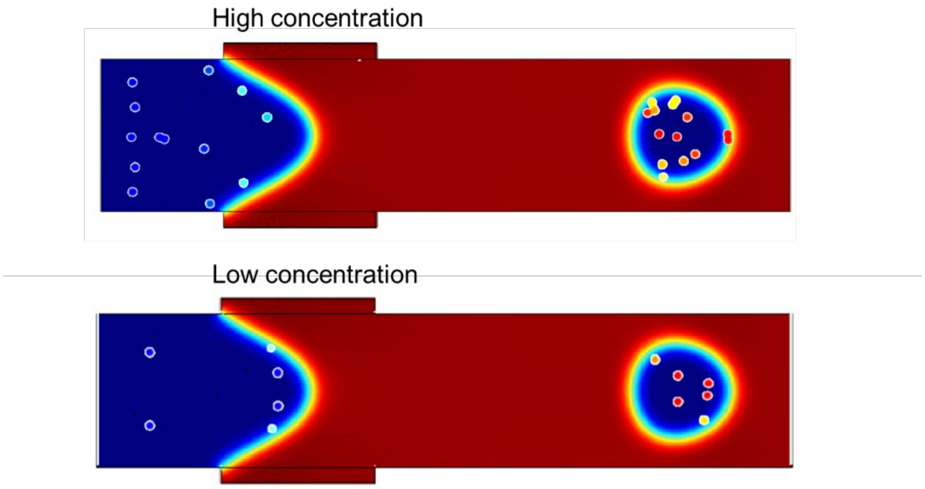


Figure S3. Simulation of droplet formation using different protein concentrations.

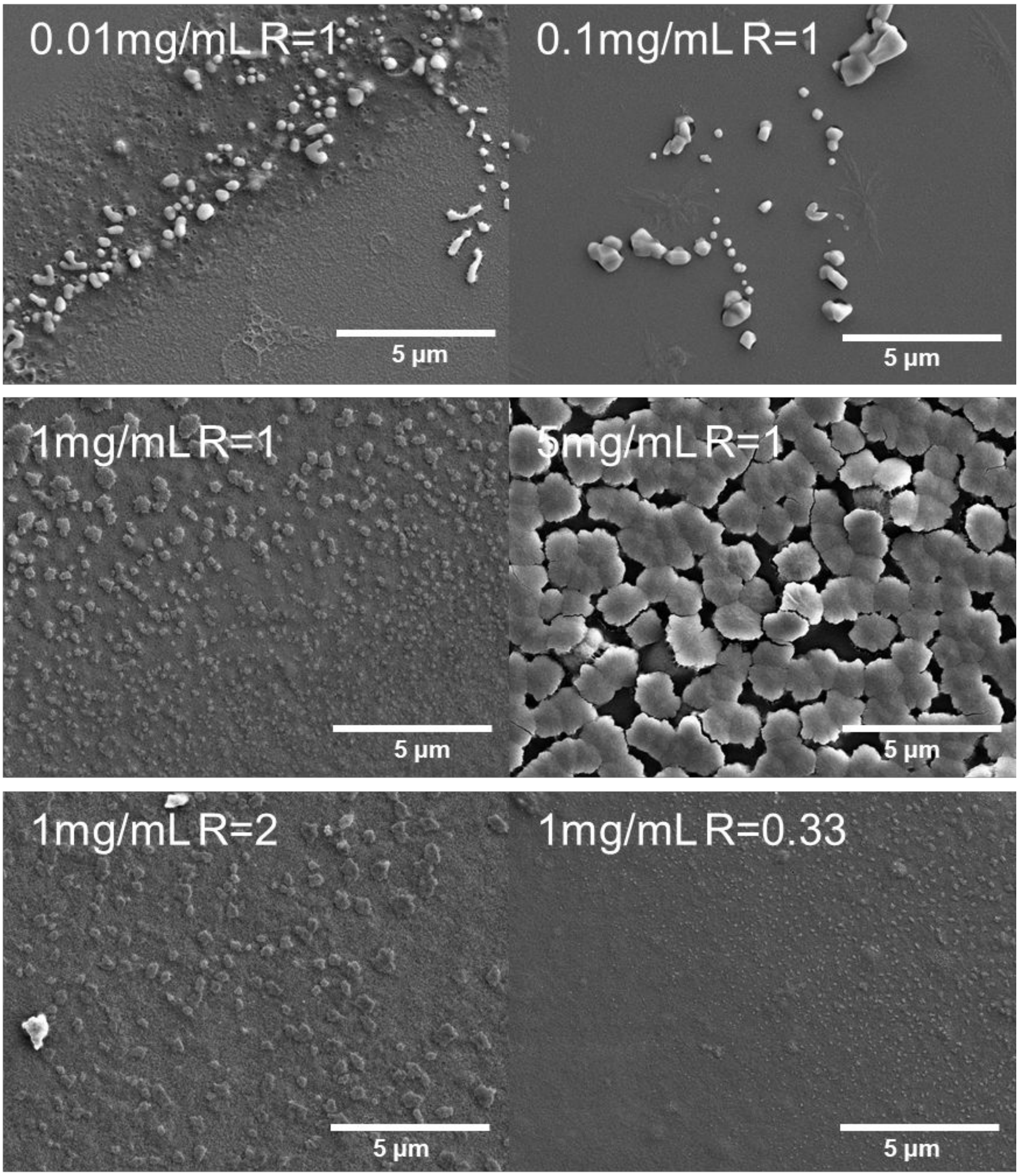


Figure S4. The SEM micrographs of a bulk method of forming protein nanoparticles
Particle size ranges from dozens of nanometers to several micrometers, while the morphology is irregular, and the sample is extremely polydisperse. The ethanol-to-protein ratio is denoted by R.

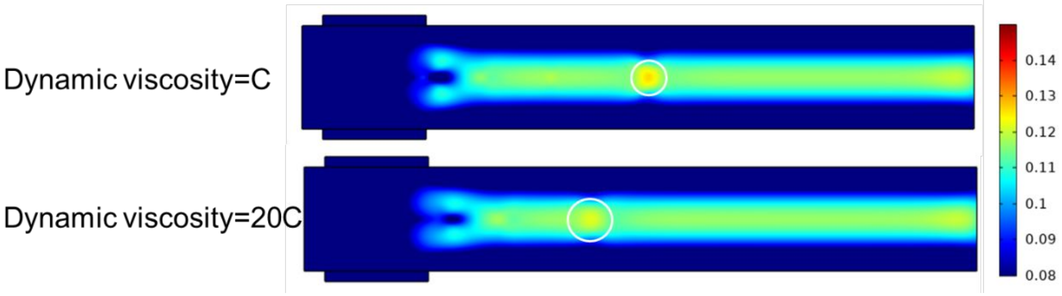


Figure S5. Simulation result showing the velocity distribution of a droplet under different dynamic viscosities. The white circle represents the droplet shape.

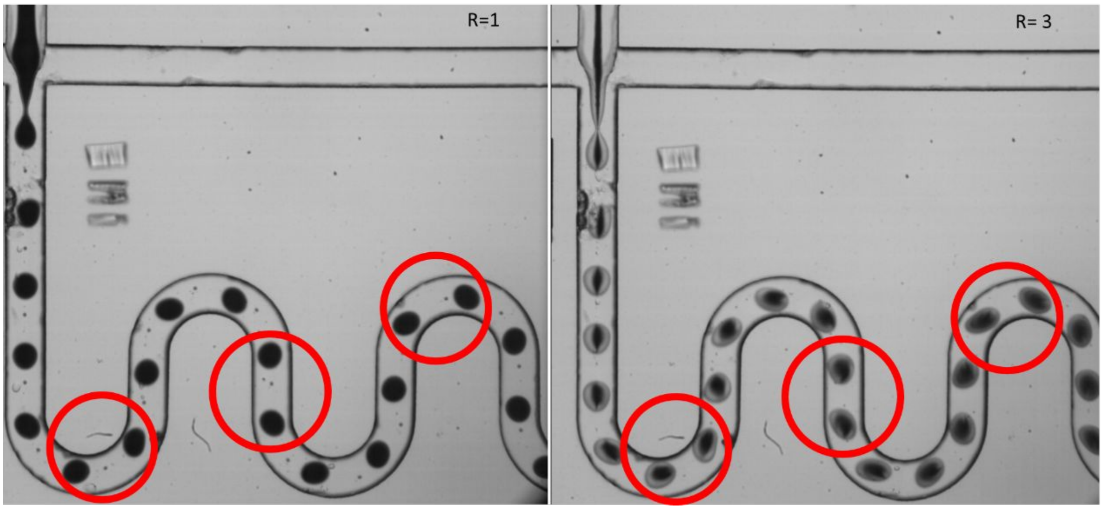

Figure S6. High-speed images of droplet formation for different ethanol-to-protein flow rate ratio systems.

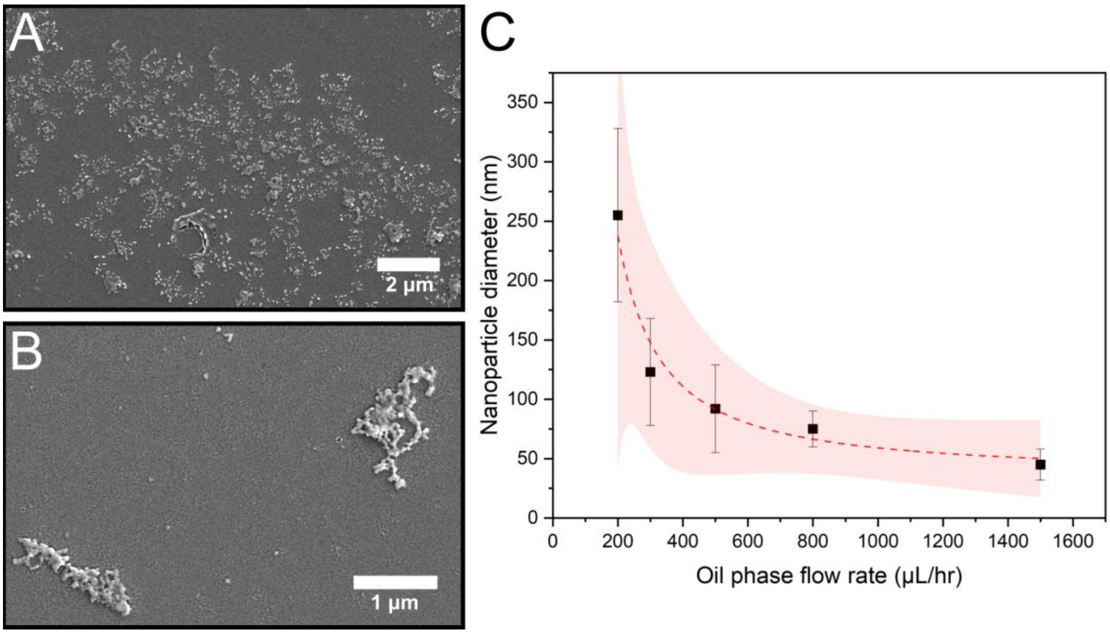

Figure S7. SEM micrographs of beta-lactoglobulin nanoparticles formed using varying oil phase flow rates. Corresponding graph of nanoparticle size as a function of the oil phase flow rate. The protein concentration used was 1 mg/mL.

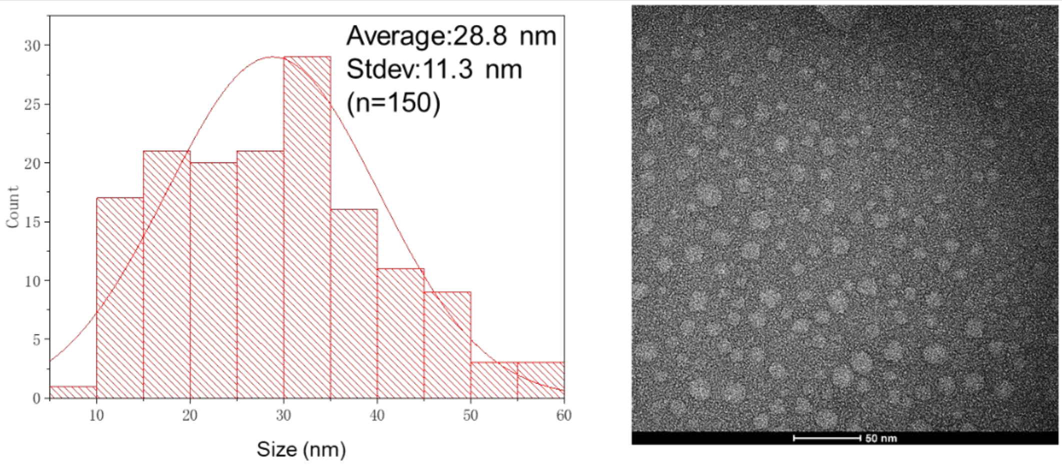


Figure S8. Protein nanoparticles obtained from the optimal conditions. Left panel: bar graph of the size distribution. Right panel: TEM micrograph of the corresponding sample.

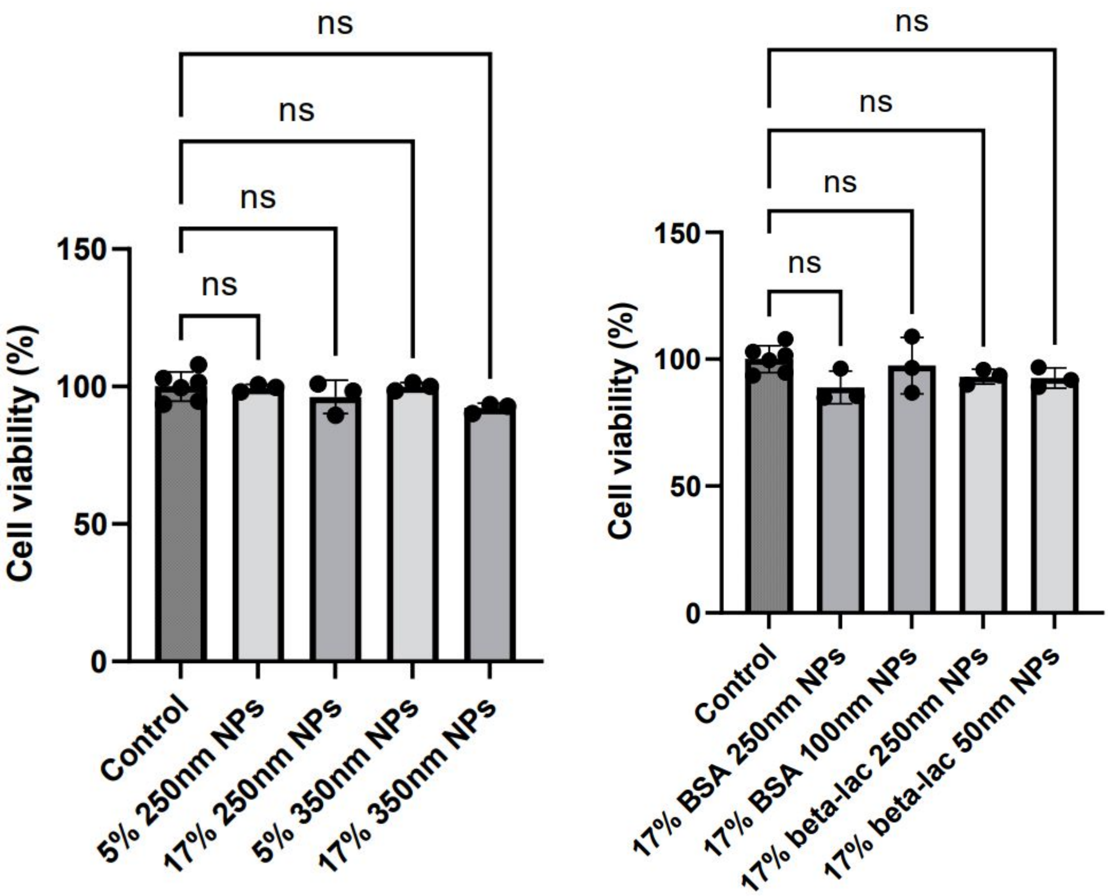


Figure S9. MTT biocompatibility results of different sized silk, BSA and beta-lactoglobulin nanoparticles. The data show the mean ± SEM of at least n=3 individual experiments. A one-way ANOVA test was conducted. In all cases, we did not see any significant difference in the viability between the control and the different nanoparticle samples. n.s – not significant

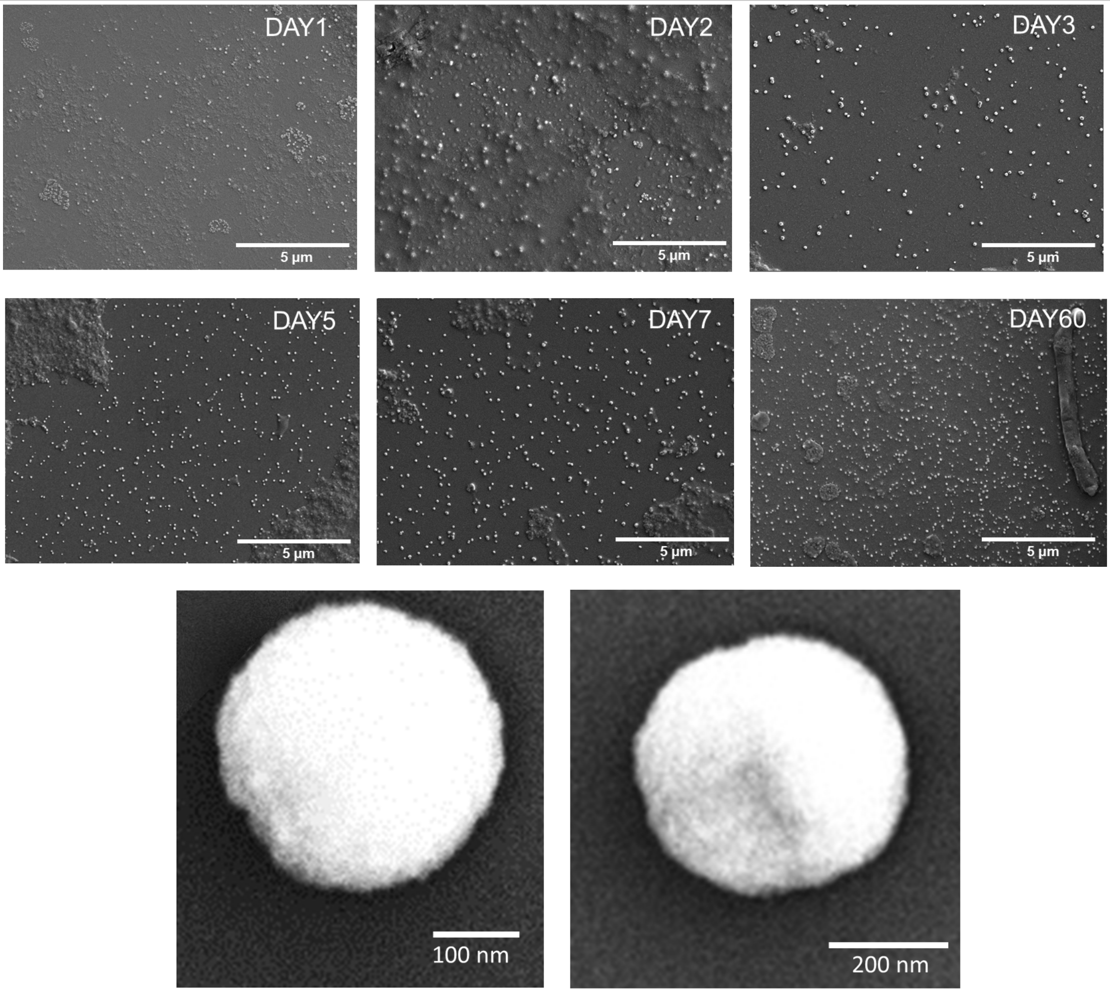


Figure S10. Nanoparticle sample analysis and corresponding SEM micrographs over a 60 day period. Following sample preparation, nanoparticle size and morphology were investigated for a total of 60 days. The SEM micrographs at the bottom two panels show a single nanoparticle. The particles exhibit a smooth and spherical morphology.

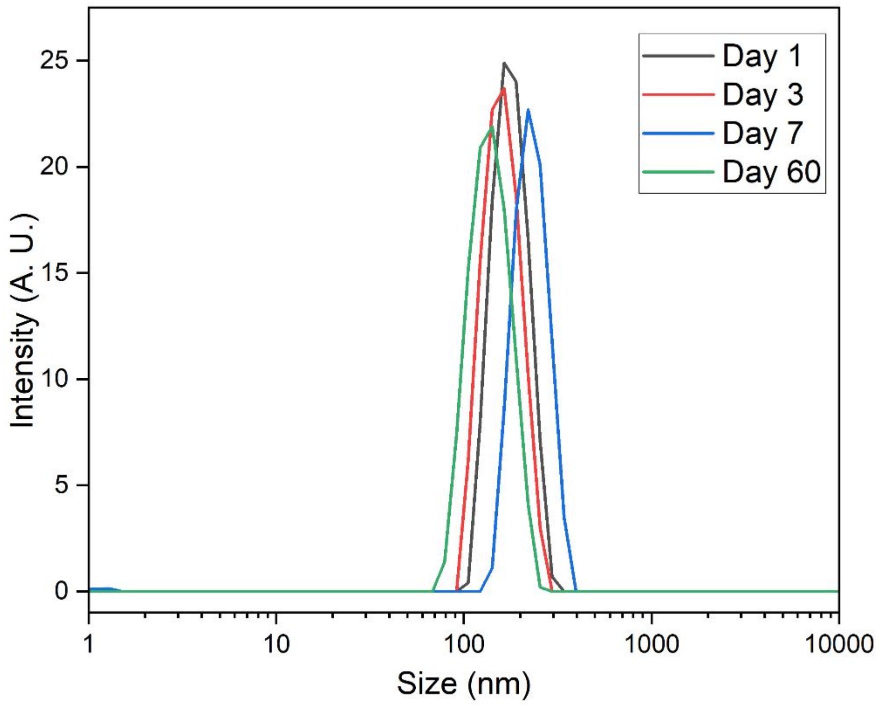


Figure S11. Dynamic light scattering (DLS) data of the nanoparticles as a function of time. Nanoparticle size was monitored for a period of up to 60 days.

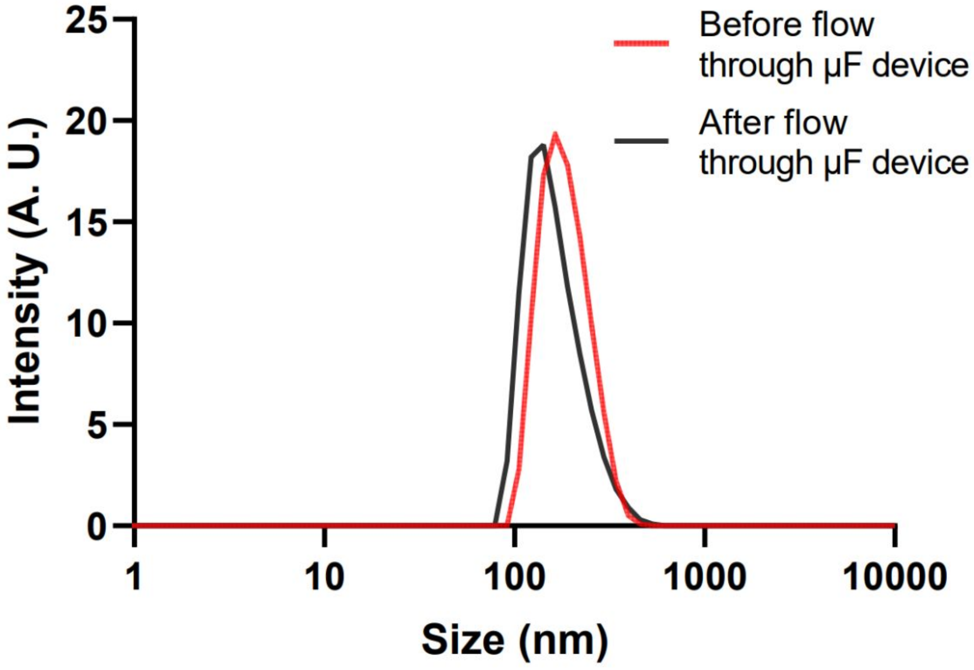


Figure S12. DLS data of the nanoparticles before and after they pass through a microfluidic channel. There is no size difference between the two samples indicating that a microfluidic environment doesn't affect nanoparticle stability.
